# Analysis of a novel calcium auxotrophy in *Aspergillus nidulans*

**DOI:** 10.1016/j.fgb.2010.04.002

**Published:** 2010-07

**Authors:** Helen Findon, Ana-Maria Calcagno-Pizarelli, José L. Martínez, Anja Spielvogel, Ane Markina-Iñarrairaegui, Tanya Indrakumar, José Ramos, Miguel A. Peñalva, Eduardo A. Espeso, Herbert N. Arst

**Affiliations:** a Department of Microbiology, Imperial College London, Flowers Building, Armstrong Road, London SW7 2AZ, United Kingdom; b Departamento de Microbiología, Campus de Rabanales, Universidad de Córdoba, E-14071 Córdoba, Spain; c Department of Cellular and Molecular Medicine, Centro de Investigaciones Biológicas, CSIC, Ramiro de Maeztu 9, Madrid 28040, Spain; d Department of Microbiology and Genetics, University of Technology Berlin, TIB 4/4-1, Gustav-Meyer-Allee 25, D-13355 Berlin, Germany

**Keywords:** Aspergillus, Calcium transport, Cation homeostasis, Ion pump, Vacuolation, Transcriptional regulation

## Abstract

In *Aspergillus nidulans* a combination of null mutations in *halA*, encoding a protein kinase, and *sltA*, encoding a zinc-finger transcription factor having no yeast homologues, results in an elevated calcium requirement (‘calcium auxotrophy’) without impairing net calcium uptake. *sltA*^−^ (±*halA*^−^) mutations result in hypertrophy of the vacuolar system. In *halA*^−^*sltA*^−^ (and *sltA*^−^) strains, transcript levels for *pmcA* and *pmcB*, encoding vacuolar Ca^2+^-ATPase homologues, are highly elevated, suggesting a regulatory relationship between vacuolar membrane area and certain vacuolar membrane ATPase levels. Deletion of both *pmcA* and *pmcB* strongly suppresses the ‘calcium auxotrophy’. Therefore the ‘calcium auxotrophy’ possibly results from excessive vacuolar calcium sequestration, causing cytosolic calcium deprivation. Null mutations in *nhaA*, homologous to *Saccharomyces cerevisiaeNHA1*, encoding a plasma membrane Na^+^/H^+^ antiporter effluxing Na^+^ and K^+^, and a non-null mutation in *trkB*, homologous to *S. cerevisiaeTRK1*, encoding a plasma membrane high affinity K^+^ transporter, also suppress the calcium auxotrophy.

## 1 Introduction

The biological importance and pervasive roles of calcium have spawned numerous investigations in a plethora of organisms. Yet there remains a need for new tools and novel approaches if calcium homeostasis and signalling are to be thoroughly understood. One severe difficulty for investigating the roles of calcium is that many microorganisms, including the model filamentous ascomycete fungus *Aspergillus nidulans*, require only minute quantities of calcium, easily satisfied by trace contamination of media components. The literature contains very few examples of elevated calcium requirements or ‘calcium auxotrophies’ and the degree to which they have been exploited is limited. In *Saccharomyces cerevisiae* loss of the Golgi Ca^2+^-ATPase Pmr1p impairs Golgi function unless high Ca^2+^ levels are supplied exogenously (Antebi and Fink, 1992). Although double mutants lacking both Pmr1p and the vacuolar membrane Ca^2+^-ATPase Pmc1p are inviable irrespective of Ca^2+^ concentration, triple mutants also defective in the Ca^2+^-binding subunit of calcineurin Cnb1p are viable but require exogenous Ca^2+^ for growth (Cunningham and Fink, 1994; Sze et al., 2000). These *pmr1 pmc1 cnb1* triple mutants are hypersensitive to high Na^+^ levels but this salt hypersensitivity can be overcome by heterologous expression of an endoplasmic reticulum-located Ca^2+^-ATPase (Anil et al., 2008). In any consideration of calcium homeostasis, it is also important to remember that fungal vacuoles are a major site of Ca^2+^ storage (Dunn et al., 1994; Klionsky et al., 1990).

Here we report a new genetic approach using *A. nidulans*, a mutationally based elevated requirement for calcium or ‘calcium auxotrophy’, enabling calcium deprivation conditions and resulting from mutations affecting gene expression rather than components of the calcium homeostasis and signalling systems. Specifically, null mutations in *halA*, when combined with null mutations in *sltA,* result in an elevated requirement for calcium. *halA* (Espeso et al., 2005) is a homologue of *S. cerevisiae HAL4* and *HAL5,* which encode redundant protein kinases necessary for cation tolerance (Mulet et al., 1999) and involved in stabilizing the major high affinity K^+^ transporter along with a number of other transporters at the plasma membrane (Pérez-Valle et al., 2007), whereas *sltA* (O’Neil et al., 2002; Spathas, 1978; Spielvogel et al., 2008) encodes a zinc-finger transcription factor necessary for tolerance of a number of cations other than calcium (Spielvogel et al., 2008), which is homologous to the Ace1 transcriptional repressor of cellulase and xylanase genes of *Trichoderma reesei* (Saloheimo et al., 2000). In contrast to *halA*, *sltA* has no identifiable homologue in *S. cerevisiae* and other yeasts, suggesting that it plays a role specific to filamentous fungi.

This double mutation-based ‘calcium auxotrophy’ allows direct selection of suppressor mutations as well as testing of potential suppressor mutations obtained by reverse genetics. Such suppressor mutations can identify other calcium-related genes and here they point to relationships between calcium and vacuolation and between alkali metal cation homeostasis and that of calcium.

## 2 Materials and methods

### 2.1 A. nidulans techniques

*A. nidulans* strains carried previously described markers and were constructed and genetically characterised by standard techniques (Arst et al., 1979; Clutterbuck, 1974, 1993; Espeso et al., 2005; Nayak et al., 2006; Spielvogel et al., 2008) except for *gfp::pepA* (AN4416) and deletion alleles of *pmcA* (AN1189), *pmcB* (AN4920), *trkA* (AN5636), *trkB* (AN8029) and *vcxA* (AN0471) which were constructed by standard techniques (Araújo-Bazán et al., 2009; Szewczyk et al., 2006; Taheri-Talesh et al., 2008; Yu et al., 2004) (also see Supplementary Table S1). *halA24* and *sltA1* have null phenotypes (Espeso et al., 2005; Spielvogel et al., 2008). Standard minimal (MM) and complete (CM) media (Cove, 1966) were used. *trkB1* and *nhaA* mutations were selected after UV mutagenesis in a strain of genotype *pabaA1 yA2* *halAΔ::pyr-4* *sltAΔ::riboB^f^* (where *pyr-4* is the *Neurospora crassa pyrG* orthologue and *riboB^f^* is the *Aspergillus fumigatus riboB* orthologue) as allowing much improved growth on supplemented minimal medium containing no added calcium and 80 μM BAPTA [1,2-bis(2-aminophenoxy)ethane-N,N,N′,N′-tetraacetic acid] after 5 days at 37 °C. The full genotypes of strains used in displayed experiments are listed in Supplementary Table S2. All mutant alleles obtained in this work were subjected to extensive genetic analysis and strains used in displayed experiments were typical of strains having the same relevant genotype, i.e. no genetic background effects were observed.

The strains used for transformation had genotypes HHF24a (*pabaA1* *yA2* *argB2* *inoB2* *pyroA4* *nkuAΔ*::*bar* *niiA4*) for *pmcA* and *pmcB* deletions, HHF24c (*pabaA1* *yA2* *inoB2* *pyroA4* *nkuAΔ*::*bar* *niiA4*) for *trkA* deletion, MAD1732 (*pyrG89* *pabaA1* *inoB2* *wA4* *pacC900* *nkuAΔ*::bar) for *trkB* deletion, MAD1739 (*pyrG89* *pyroA4* *nkuAΔ*::*bar*) for *vcxA* deletion and AMC56 (*pyrG89* *biA1* *pyroA4* *nkuAΔ*::*bar*) for *gfp*::*pepA* tagging.

### 2.2 Alkali metal cation transport

Cation content measurements were as described with minor modifications (Cabello-Hurtado et al., 2000; Ramos and Rodríguez-Navarro, 1985). For Rb^+^ uptake, 10^6^ spores were inoculated into 100 ml of K^+^-free minimal MM (with ${\text{NH}}_{4}^{+}$ as sole cation) and incubated at 28 °C and 150 rpm. overnight. Initially, 25 ml of culture was removed, dried, weighed and used to calculate dry weight. Then, flasks were placed in a shaking bath at 37 °C and RbCl (800 μM) was added. At intervals, 5 ml samples were filtered and washed in order to follow rubidium uptake. The cell-containing filters were acid-extracted overnight. For Li^+^ efflux, culture conditions were the same as for Rb^+^ uptake, but the (initially K^+^-free) MM was supplemented with KCl and LiCl (10 mM each). After dry weight determination, cells were collected and resuspended in Li^+^- and K^+^-free minimal medium. Flasks were placed in a bath at 37 °C and 5 ml samples were filtered and extracted at different times. Rb^+^ and Li^+^ contents were determined by atomic absorption spectrophotometry.

### 2.3 Ca^2+^ transport

Largely following (Ugalde and Pitt, 1986), mycelia were grown 18 h at 37 °C in supplemented MM with 10 mM CaCl_2_. Mycelia were harvested and 5 g mycelia were then incubated in 250 ml of Ca^2+^-free MM for an additional hour. Calcium transport was measured using 25 ml of this culture (0.5 g mycelium) under aeration conditions (2 l min^−1^) in MM containing 1 μCi/ml (640 pmol/ml, 640 nM) ^45^Ca^2+^ with or without cold CaCl_2_. One milliliter samples were taken at indicated times, washed in Ca^2+^-free MM containing 100 μM BAPTA and resuspended in scintillation liquid. The maximum incorporation measured was of 270 pmol ^45^Ca^2+^ per ml of mycelial suspension, containing approx. 3.5 mg of dry mycelia. The graph shows the amount of ^45^Ca^2+^ taken up in pmol/mg of dried mycelia.

### 2.4 Microscopy

*A. nidulans* cells were cultured at 25 °C in watch minimal medium as described (Peñalva, 2005). Z-series stacks were deconvolved using Huygens essential software (http://www.svi.nl/). Maximal projections of z-series stacks were made with Volocity (http://www.improvision.com) or ImageJ (http://rsb.info.nih.gov/ij/) software. About 40–70 μm germlings were divided into three regions: basal (50%), medial (25%) and tip (25%). Vacuole measurements were made with Volocity software; vacuoles were visualised using CellTracker^TM^ CMAC blue (7-amino-4-chloromethycoumarin) and the largest vacuoles in medial regions were measured. Staining with FM4-64 and CMAC blue was as described (Abenza et al., 2009; Peñalva, 2005). Germlings were cultured using uncoated, glass bottom culture dishes (MatTek Corporation). Fluorescence images were acquired using Hamamatsu ERG cameras and Zeiss Axiovert 200 or Leica DMI600 inverted microscopes (63×, 1.4 numerical aperture objectives), using Zeiss or Semrock specific filter sets.

### 2.5 Construction of deletion cassettes and GFP tagging of PepA

Gene deletion followed the protocol essentially as described (Araújo-Bazán et al., 2009). Primers are described in Supplementary Table S1. The *A. fumigatus* genes used were: *pyroA* (AN1189/*pmcA*), *pyrG* (AN0471/*vcxA*, AN8029/*trkB*), and *pabaA* (AN5636/*trkA*, AN4920/*pmcB*). Fusion PCR products were purified and used to transform *nkuAΔ* strains. Transformants were selected on media lacking the supplement for the auxotrophic marker and the deletion of the target open reading frame was confirmed by both Southern blot and PCR analysis.

The t-SNARE (soluble N-ethylmaleimide-sensitive factor attachment protein receptor present in the ‘target’ membrane in heterotypic membrane fusion events) Pep12p homologue, PepA (AN4416), was tagged with GFP at its N-terminus using a modified fusion PCR protocol of (Taheri-Talesh et al., 2008; Yang et al., 2004; Yu et al., 2004), with *A. fumigatus pyrG* as selection marker (primers in Supplementary Table S1). Five transformants showed tandem integration and one multiple integration. The six strains gave identical localisation patters for the tagged *A. nidulans* Pep12p homologue. One transformant with tandem integration was selected for subsequent use. The *sltAΔ* strain expressing GFP-PepA, AMC289 (Supplementary Table S2), was constructed by meiotic recombination.

### 2.6 Real time quantitative-PCR (RTQ-PCR)

3 × 10^6^ conidiospores ml^−1^ were inoculated into flasks containing 100 ml minimal medium to which no calcium had been added. Cultures were incubated for 16 h at 30 °C in a shaking incubator at 200 rpm. Mycelia were harvested by filtration and frozen at −80 °C. RNA was extracted using RNA-Bee (AMS Biotechnology). A 125 ng of total RNA was used to synthesise cDNA using an archive kit (Applied Biosystems). One μl of cDNA was used with PCR master mix and TaqMan (Applied Biosystems) in an Applied Biosystems 7500 PCR system, following the manufacturer’s instructions. Cycle thresholds were normalised to 18S rRNA and expressed relative to wild type. Probes are listed in Supplementary Table S1.

### 2.7 Statistical analysis

GraphPad Prism 5 software (http://www.graphpad.com/prism/Prism.htm) and the Newman-Keuls Multiple Comparison Test were used. Use of Tukey’s multiple comparison test gave very similar results.

## 3 Results

### 3.1 The combination of halA^−^ and sltA^−^ mutations results in an elevated requirement for calcium

**Fig. 1:**
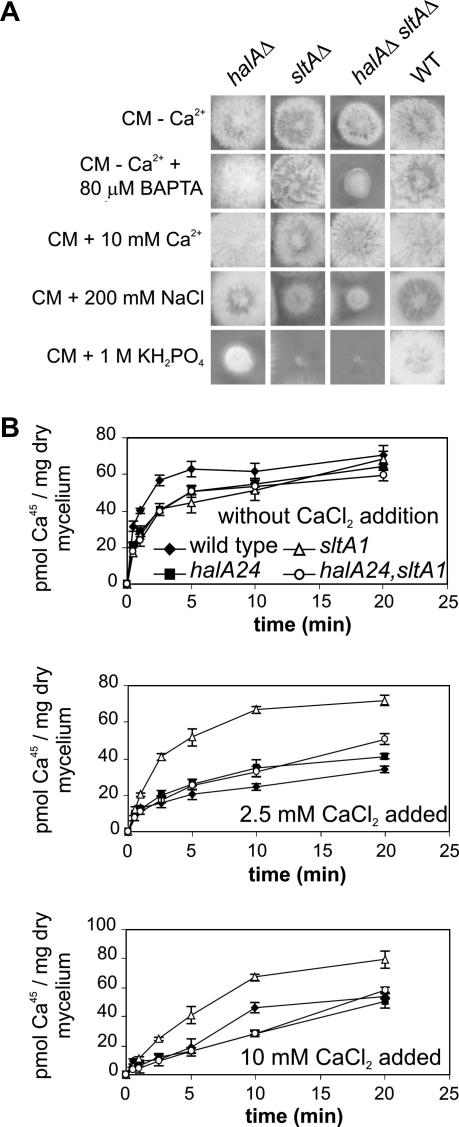
The calcium auxotrophy and effects of *halA* and *sltA* mutations on calcium transport. (A) Growth of strains having the indicated relevant genotypes after 2 days on appropriately supplemented CM at 37 °C with no added calcium with or without 80 μM BAPTA or 200 mM NaCl or 1 M KH_2_PO_4_ or with 10 mM CaCl_2_ is shown. Virtually identical results were obtained when KCl or NaH_2_PO_4_ were used at the same concentrations as NaCl and KH_2_PO_4_ and the growth conditions shown were chosen to show that Na^+^ and K^+^ are toxic to similar extents and that the toxicity is independent of the anion present. (B) ^45^Ca^2+^ uptake was followed for 20 min in wild type, *halA24*, *sltA1* and *halA24 sltA1* strains with addition of 0, 2.5 or 10 mM cold CaCl_2_. Error bars indicate s.d.; *N* = 3.

*A. nidulans* is extremely salt tolerant (e.g. able to grow on solid medium saturated with NaCl). A previously unreported aspect of the phenotype of null *halA* mutations in *A. nidulans* is poor growth in the presence of high concentrations (e.g. 1 M) of Na^+^ or K^+^ (Fig. 1A). In contrast to *hal4 hal5* double mutants of *S. cerevisiae* (Mulet et al., 1999), null *halA* mutants are not abnormally sensitive to high Ca^2+^ concentrations. Null mutations in *sltA* result in even more extreme Na^+^/K^+^ sensitivity than null *halA* mutations (O’Neil et al., 2002; Spathas, 1978; Spielvogel et al., 2008) (Fig. 1A). Therefore, to test additivity of alkali cation sensitivities, we constructed *halA*^−^ *sltA*^−^ double mutants. Not only are the mutations additive, resulting in extreme sensitivity to a number of cations, both divalent and monovalent, but the double mutants grow poorly on standard minimal and complete media. However, a striking exception to this extreme cation sensitivity is Ca^2+^. Ca^2+^ and, to a lesser extent, Sr^2+^ substantially improve growth of the double mutants on minimal and complete media such that we routinely supplement them with Ca^2+^ (Fig. 1A). The calcium chelator BAPTA exacerbates the poor growth of *halA*^−^ *sltA*^−^ strains on media lacking added Ca^2+^ (Fig. 1A).

### 3.2 The calcium auxotrophy does not result from defective calcium transport

The most obvious basis for an elevated requirement for calcium would be a defect in bulk net calcium transport. However, whether grown with 0, 2.5 or 10 mM Ca^2+^, *halA*^−^ *sltA*^−^ strains are not defective in bulk net ^45^Ca^2+^ uptake (Fig. 1B). It is, however, worth noting that calcium-grown mycelia of the *sltA*^−^ strain, but not the *halA*^−^ *sltA*^−^ strain, show elevated calcium uptake and that, in contrast to the other three strains, the *sltA*^−^ strain shows no indication that unlabelled calcium is competing with the labelled calcium (Fig. 1B). In other words, calcium uptake does not appear saturable in the *sltA*^−^ mutant.

### 3.3 sltA^−^ mutations result in hypertrophy of the vacuolar system

**Fig. 2:**
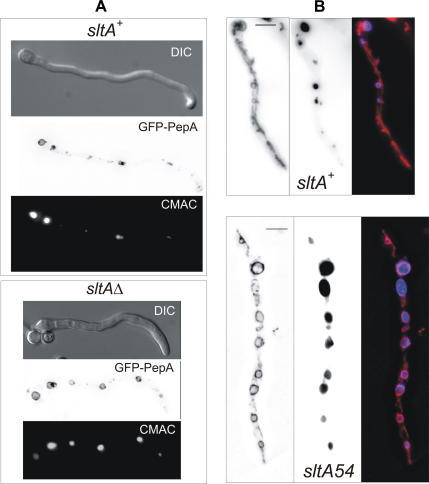
Vacuolation of *sltA* mutant and wild type strains. (A) Vacuoles were visualised using CellTracker^TM^ CMAC blue or, in confirmation, the endosomal and (mainly) vacuolar membrane-targeted GFP-tagged t-SNARE PepA (homologue of *S. cerevisiae* Pep12p). The large vacuole at the left for each strain is the basal conidiospore vacuole. DIC = differential interference contrast. (B) Vacuoles were visualised with CMAC blue and germlings were stained with FM4-64 with the merged image also shown. *sltA54* is a partial loss-of-function mutation whose selection and characterisation will be described elsewhere. Bar = 5 μ.

**Fig. 3:**
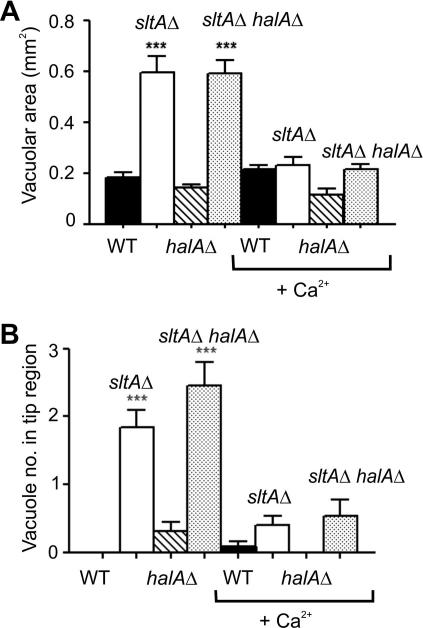
Quantitation of vacuolar system hypertrophy in *sltAΔ* strains. (A) Vacuolar areas of maximum projection in *halAΔ*, *sltAΔ*, *halAΔ sltAΔ* and wild type medial regions of hyphae grown with or without 10 mM CaCl_2_. Error bars indicate s.d.; ^***^ indicates *p* < 0.001 as compared to wild type (for *sltAΔ*) and to *halAΔ* (for *halAΔ sltAΔ*) grown in the same conditions. *N* ⩾ 10. (B) Numbers of vacuoles in tip regions of *halAΔ*, *sltAΔ*, *halAΔ sltAΔ* and wild type germlings grown with or without 10 mM CaCl_2_. Statistical analysis as in (A). *N* ⩾ 10.

**Fig. 4:**
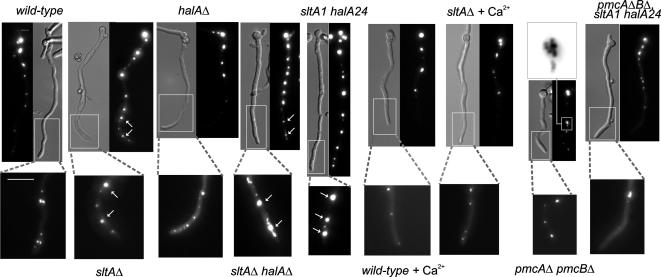
The effects of Ca^2+^ and *pmcAΔ pmcBΔ* on vacuolation. Vacuoles visualised using CMAC blue in strains of the indicated relevant genotypes are shown in white. Where indicated, strains were grown in the presence of 15 mM CaCl_2_.

As bulk calcium transport is not deficient in the calcium auxotrophs, our attention turned to the intracellular distribution of calcium and in particular to the role of vacuoles. CMAC blue has previously been used successfully to visualise vacuoles in both *Aspergillus oryzae* (Tatsumi et al., 2007) and *A. nidulans* (Apostolaki et al., 2009) and therefore we were able to follow established protocols. In addition, to confirm vacuolar identity, we used a GFP-tagged version of the endosomal and (mainly) vacuolar membrane-targeted t-SNARE PepA, the orthologue of *S. cerevisiae* Pep12p (our unpublished results). In *A. nidulan*s wild type germlings, vacuoles increase in volume with the relative distance to the tip, such that the largest vacuole is usually located in the basal conidiospore (Abenza et al., 2009). In comparison, vacuoles are generally much larger and extend much further toward the tips in *sltA* mutants (Fig. 2). The area of maximum projection of the largest *sltAΔ* medial vacuoles [CMAC blue-staining, >35 nm diameter circular structures] is approximately threefold greater than that in wild type (Fig. 3A). If vacuoles are assumed spherical for simplification, a threefold area difference would equate to a greater than fivefold difference in volume. Fig. 3B shows quantitatively the differences in vacuole numbers in hyphal tip regions between a *sltA* null mutant and wild type. In wild type the lipophilic dye FM4-64 reaches mitochondrial and endoplasmic reticulum as well as vacuolar membranes (Peñalva, 2005), but in a *sltA* mutant it preferentially accumulates at vacuolar membranes (Fig. 2B). Growth in the presence of calcium largely alleviates vacuolar system hypertrophy (Figs. 3 and 4).

### 3.4 Lack of two putative vacuolar Ca^2+^-ATPases results in vacuolar fragmentation and suppresses the calcium auxotrophy

**Fig. 5:**
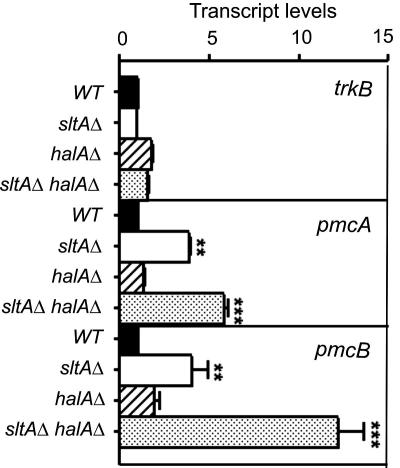
Relative transcript levels for *trkB*, *pmcA* and *pmcB* as determined by real time quantitative-PCR in *halAΔ*, *sltAΔ*, *halAΔ sltAΔ* and wild type strains. Error bars indicate s.d.; duplicate biological samples were used with 3 replicates each. ^***^ Indicates *p* < 0.001 and ^**^ indicates *p* < 0.01.

**Fig. 6:**
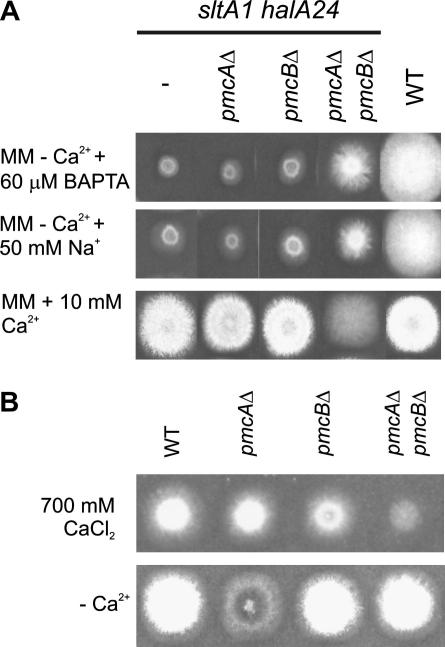
Suppression of the calcium auxotrophy by *pmcAΔ pmcBΔ* and very slightly by *pmcBΔ* and calcium inhibition of *pmcAΔ pmcBΔ* double mutants. (A) Growth of strains having the indicated relevant genotypes after 2 days at 37 °C on appropriately supplemented MM containing 60 μM BAPTA, 50 mM NaCl or 10 mM CaCl_2_ is shown. (B) Calcium inhibition of *pmcAΔ pmcBΔ* double mutants. Growth of strains having the indicated relevant genotypes after 2 days at 37 °C on appropriately supplemented MM with or without 700 mM CaCl_2_ is shown.

In view of the hypervacuolation of calcium auxotrophs, we investigated levels of putative vacuolar calcium pumps to see whether excessive vacuolar calcium storage might be an issue. A striking effect of deletion of *sltA* is an increase in *pmcA* (AN1189) and *pmcB* (AN4920) transcript levels (Fig. 5). *pmcA* and *pmcB* are homologues of *S. cerevisiae PMC1*, encoding a vacuolar membrane Ca^2+^-ATPase responsible for pumping excess cytosolic calcium into vacuoles (Cunningham and Fink, 1994; Marchi et al., 1999). These transcript level increases are greater still if *halA* is also deleted, reaching twelve-fold for *pmcB* and sixfold for *pmcA* (Fig. 5). Deletion of *pmcB* weakly suppresses and deletion of both *pmcA* and *pmcB* strongly suppresses the calcium auxotrophy (Fig. 6A). Suppression is independent of BAPTA as it occurs when Na^+^ is used to reduce residual growth due to trace calcium contamination and when neither BAPTA nor Na^+^ is added (Fig. 6A). Deletion of both *pmcA* and *pmcB* also confers a very slight level of Na^+^ tolerance to *halA*^−^ *sltA*^−^ strains although they remain extremely Na^+^ sensitive. The deletion of both *pmcA* and *pmcB* additionally results in vacuolar fragmentation and such fragmentation occurs irrespective of the *halA* and *sltA* alleles carried (Fig. 4, inverted contrast inset). Very high Ca^2+^ concentrations (e.g. 700 mM) are moderately toxic to the wild type, slightly more toxic to *pmcAΔ* and *pmcBΔ* strains and very toxic to *pmcAΔ pmcBΔ* double mutants (Fig. 6B). Even in the absence of exogenously added Ca^2+^, the growth of *pmcAΔ pmcBΔ* strains can be improved by addition of Na^+^ or BAPTA to prevent inhibition by trace calcium contamination. Inability of the *halA*^−^ *sltA*^−^ combination to ameliorate calcium toxicity in *pmcAΔ pmcBΔ* strains is further evidence that the calcium auxotrophy does not result from reduced calcium uptake. We should note that two Pmc1p homologues localise to both the vacuolar and plasma membranes in *N. crassa* (Bowman et al., 2009) but caution should be exercised in the interpretation of these results as the tagged proteins were expressed using a heterologous promoter and might have been expressed at higher than physiological levels.

Deletion of *vcxA* (Spielvogel et al., 2008), the homologue of *S.* *cerevisiae VCX1*, encoding a vacuolar membrane Ca^2+^/H^+^ exchanger removing excess cytosolic calcium (Miseta et al., 1999; Pozos et al., 1996) does not suppress the calcium auxotrophy. However, although *sltA*^−^ strains do overexpress *vcxA*, such elevated expression is strictly dependent upon added Ca^2+^ (Spielvogel et al., 2008).

### 3.5 Mutations in genes involved in alkali metal cation transport can suppress the calcium auxotrophy

**Fig. 7:**
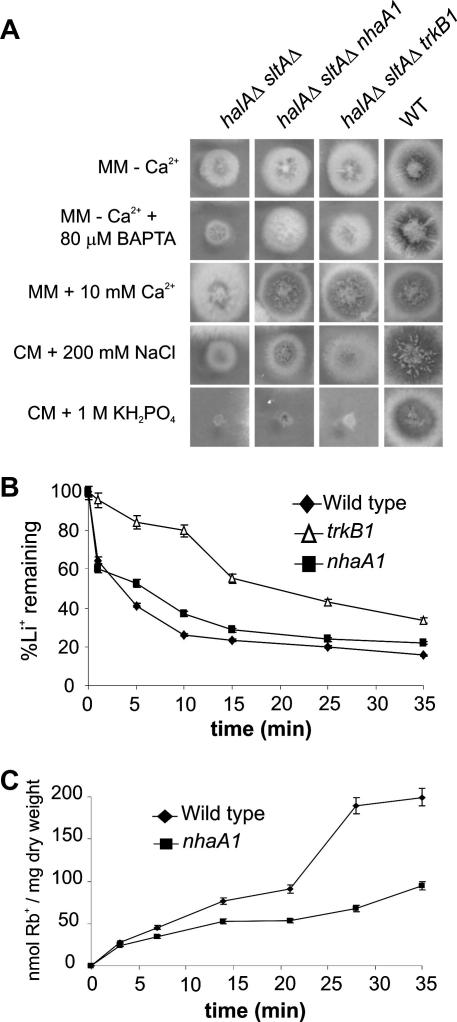
Suppression of the calcium auxotrophy by *nhaA1* and *trkB1* and the effects of these mutations on alkali metal cation transport. (A) Growth of strains having the indicated relevant genotypes after 2 days at 37 °C on appropriately supplemented MM with 10 mM CaCl_2_ or no added calcium with or without 80 μM BAPTA or on appropriately supplemented CM with either 200 mM NaCl or 1 M KH_2_PO_4_ is shown. (B) Efflux of pre-loaded Li^+^ by *trkB1* and *nhaA1* strains. Error bars indicate s.d.; *N* ⩾ 3. (C) Rb^+^ uptake by a *nhaA1* strain grown in limiting potassium medium. The *trkB1* mutant fails to grow in limiting potassium medium. In mycelia grown in potassium-replete medium, Rb^+^ uptake levels are much lower and do not differ appreciably among these three strains. Error bars indicate s.d.; *N* ⩾ 3.

**Fig. 8:**
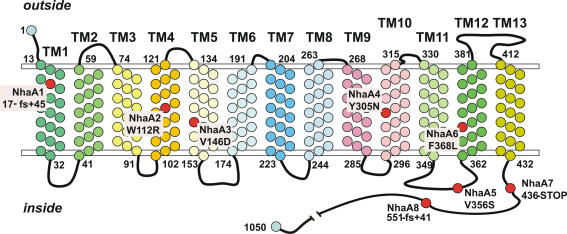
A predicted topology of the NhaA protein and the positions and amino acid sequence changes of the mutations sequenced. fs = frame-shift.

In order to see whether there might be other mechanisms for alleviating the calcium auxotrophy in addition to elimination of vacuolar calcium pumps, we sought suppressor mutations using classical genetics methodology. Mutations able to suppress the elevated calcium requirement of a *halAΔ sltAΔ* strain were selected on medium containing BAPTA but no added calcium after ultraviolet mutagenesis (Fig. 7A). Given that suppression is evident in both the absence and presence of BAPTA, the suppressors clearly do not act through an effect on BAPTA. Minor phenotypic differences suggested that the suppressor mutations fell into two classes. Using a laborious combination of parasexual, mitotic recombination and sexual analyses, all in *halA*^−^ *sltA*^−^ backgrounds, genetic map positions were determined for representatives of both mutant classes. The mutated genes were determined to be in centromere-distal regions of the left arms of chromosomes II and IV, respectively. Plausible candidate autocalled genes in the relevant genomic regions were then sequenced until mutational changes were identified. Of the nine suppressor mutations sequenced, eight are in *nhaA* (AN7250) (Fig. 8), a homologue of *S. cerevisiae NHA1*, encoding a plasma membrane Na^+^/H^+^ antiporter involved in Na^+^ and K^+^ efflux (Bañuelos et al., 1998; Prior et al., 1996). The ninth suppressor mutation is in *trkB* (AN8029), a homologue of *S. cerevisiae TRK1*, encoding a plasma membrane high affinity K^+^ transporter (Gaber et al., 1988; Ko and Gaber, 1991). There are three *A. nidulans* homologues of the *S. cerevisiae* plasma membrane high affinity K^+^ transporters Trk1p and Trk2p. TrkB and TrkA (AN5636) are more closely related to Trk1p whereas TrkC (AN10136) is more closely related to Trk2p. *nhaA1* creates a frameshift in codon 17, showing that null *nhaA* mutations can suppress the calcium auxotrophy. In contrast, *trkBΔ* does not suppress the calcium auxotrophy, thus establishing that the *trkB1* mutation, resulting in Ser372Leu in the 829 residue TrkB protein, is not a null mutation, a conclusion supported by the fact that the *trkB1* mutation is partially dominant in diploids. The responses of *trkB1* and *trkBΔ* strains to Li^+^, Rb^+^ and Cs^+^ toxicities are also quite different. For example, on solid minimal medium in which K^+^ is replaced by equimolar levels of Na^+^, *trkB1* strains are slightly resistant to the toxicity of 20 mM Cs^+^ whereas *trkBΔ* strains are hypersensitive. We have not investigated *trkC* but *trkAΔ* also does not suppress the calcium auxotrophy. Transcript levels for *trkB* do not differ appreciably among *halAΔ*, *sltAΔ*, *halAΔ sltAΔ* and wild type strains (Fig. 5) and transcript levels for *nhaA* in these strains are virtually undetectable. To establish firmly that NhaA and TrkB are involved in alkali metal ion transport, we examined the effects of *nhaA1* and *trkB1* on efflux of pre-loaded Li^+^, a Na^+^ surrogate, and uptake of the K^+^ surrogate Rb^+^. *trkB1* and, to a much lesser extent, *nhaA1* impair efflux of pre-loaded Li^+^ (Fig. 7B). Although *trkB1* strains fail to grow in limiting K^+^ conditions, *nhaA1* strains transport Rb^+^ at a reduced rate (Fig. 7C).

## 4 Discussion

We have shown that a combination of null mutations in *halA* and *sltA* results in an elevated calcium requirement or calcium auxotrophy, which is exacerbated by a calcium chelator and by alkali metal cations, and used a combination of reverse and classical genetics, cell biology and biochemical techniques to investigate its basis. Our finding that this calcium growth requirement is not the result of defective calcium uptake led us to investigate whether abnormal intracellular calcium distribution might be responsible. We found that *sltA*^−^ mutations result in hypertrophy of the vacuolar system and markedly increase transcript levels for two putative vacuolar calcium pumps. In *halA*^−^ *sltA*^−^ double mutants, transcript levels for these pumps are increased to an astonishing extent. We deleted the genes encoding these pumps and found that deletion of *pmcB* weakly and deletion of both *pmcA* and *pmcB* strongly alleviated the calcium auxotrophy of *halA*^−^ *sltA*^−^ double mutants. In accordance with a role for PmcA and PmcB in vacuolar calcium storage and detoxification, *pmcAΔ* strains are slightly hypersensitive to calcium toxicity and *pmcAΔ pmcBΔ* double mutants are considerably hypersensitive to calcium toxicity and, in accordance with the lack of a bulk calcium transport defect in the calcium auxotrophs, the *halA*^−^ *sltA*^−^ combination does not reduce calcium toxicity to *pmcAΔ pmcBΔ* strains. All of these findings suggest that excessive vacuolar storage of calcium, creating a cytosolic deficit, is the basis for the calcium auxotrophy. Although aequorin can be used to determine total calcium concentrations in fungi (Nelson et al., 2004), so far as we are aware, there are no versions capable of distinguishing vacuolar from cytosolic calcium nor are there calcium indicator chemicals suitable for use in filamentous fungi which can indicate the intracellular distribution of calcium.

To see whether there might be other mechanisms of calcium auxotrophy suppression we selected mutations bypassing the requirement for elevated calcium levels. This enabled us to show that mutations in two genes encoding alkali metal cation transporters can also suppress the calcium auxotrophy. Loss-of-function mutations in *nhaA* suppress along with a mutation likely to lead to gain-of-function or altered function in *trkB*. The preliminary indications are that the products of these genes might differ in physiological roles from those of their *S. cerevisiae* homologues. Determining the mechanisms of calcium auxotrophy suppression in these cases will require a thorough characterisation of the physiological roles of NhaA and TrkB and is beyond the scope of this work.

Beyond the present findings, this work has highlighted a number of areas for future attention:

### 4.1 Vacuolation

Firstly, the vacuolar system hypertrophy of *sltA*^−^ strains might suggest a relationship between *sltA* and autophagy and/or a relationship between *sltA* and vacuolar homotypic fusion. Although *S. cerevisiae* has no identifiable *sltA* homologue, mutation of a number of yeast genes results in oversized vacuoles (Raymond et al., 1992; Seeley et al., 2002). Secondly, the ability of exogenously added calcium to reverse the vacuolar system hypertrophy in *sltA*^−^ strains and the vacuolar fragmentation resulting from combined deletion of *pmcA* and *pmcB* indicate that the relationship between vacuolation and calcium warrants further investigation. Calcium stress results in vacuolar fragmentation in *S. cerevisiae* (Kellermayer et al., 2003). However, deletion of *S. cerevisiae PMC1* does not lead to vacuolar fragmentation (Kellermayer et al., 2003) and *pmc1Δ* was not identified by an *S. cerevisiae* genome-wide screen for deletions altering vacuolar morphology (Seeley et al., 2002). Vacuolar fragmentation in *A. nidulans* would appear to depend on the cytosolic calcium concentration because calcium addition is not necessary for its occurrence in *pmcAΔ pmcBΔ* strains, and cytosolic calcium would appear to regulate directly or indirectly vacuolar homotypic fusion. In *S. cerevisiae* t-SNARE docking triggers Ca^2+^ release from the vacuolar lumen and the resulting cytosolic Ca^2+^ wave is thought to elicit vacuolar fusion (Merz and Wickner, 2004; Wickner, 2002). Therefore, high cytosolic [Ca^2+^] might obscure the Ca^2+^ wave, thus preventing vacuolar fusion. Thirdly, why do calcium-grown cultures of *sltA*^−^ strains show elevated ^45^Ca^2+^ uptake levels and why is this attenuated by a *halA*^−^ mutation (Fig. 1B)? Elevated expression of *vcxA* in calcium-grown *sltA*^−^ strains (Spielvogel et al., 2008) might explain the increased calcium uptake. The fact that the *S. cerevisiae* Hal4p and Hal5p protein kinases are necessary for stabilization of a number of transporters at the plasma membrane (Pérez-Valle et al., 2007) might be relevant to the attenuation. Fourthly, how is the relationship between vacuolar membrane area and vacuolar membrane ATPases such as PmcA, PmcB and VcxA determined, given that transcripts encoding these proteins are increased in vacuolar system hypertrophy? Finally, the *A.* *nidulans* genome contains three *S. cerevisiae PMC1* homologues in addition to *pmcA* and *pmcB*, AN2827, AN5088 and AN8399 (Harris et al., 2009). Calcium auxotrophy suppression, calcium toxicity and other aspects of the physiological roles and relationships among the five *PMC1* homologues constitute yet another area of potential interest.

### 4.2 Alkali metal cations and calcium

Another intriguing avenue for future research and one with relevance in agriculture and medicine is the relationship between alkali metal ions and Ca^2+^. The literature contains a number of reports of antagonistic relationships (e.g. Anil et al., 2008; Berridge et al., 2003; Epstein, 1998; Laude and Simpson, 2009; Mendoza et al., 1994; Prasad et al., 2008). *halA*^−^ *sltA*^−^ strains are hypersensitive to alkali metal ions and their elevated calcium requirement can be suppressed by mutations in genes encoding alkali cation transporters. In addition, high Na^+^ concentrations can protect *pmcAΔ pmcBΔ* strains against Ca^2+^ toxicity. The mechanisms of calcium auxotrophy suppression by mutations in *trkB* and *nhaA* are of considerable interest as are the physiological roles of these genes themselves. Further characterisation of the roles and regulation of NhaA and TrkB and all other alkali metal cation transporters will be necessary in order to determine the basis(-es) for the suppression of the calcium auxotrophy by *trkB1* and the *nhaA* mutations. *trkB1* is likely to be an altered function or gain-of-function mutation. It is tempting to speculate that its mutationally altered residue, Ser372, might be a phosphorylation substrate, particularly as Yenush et al. (Yenush et al., 2005) have reported a physiological interaction between *S. cerevisiae* Trk1p and phosphatase Ppz1p and demonstrated *in vivo* phosphorylation of Trk1p. Although phosphorylation of Trk1p plays an activating role, there is no reason to suppose that this aspect of regulation has been conserved through several hundred million years of evolutionary divergence of *S.* *cerevisiae* and *A. nidulans*, particularly in view of the indications of functional divergence of the homologues in the two organisms. A negative role of Ser372 phosphorylation in TrkB seems plausible. Trk1p is phosphorylated by Hal4p and Hal5p (Mulet et al., 1999; Yenush et al., 2005) but the ability of *trkB1* to suppress the calcium auxotrophy of strains having a null *halA* allele would indicate that any phosphorylation of TrkB Ser372 is not done by the Hal4p/Hal5p homologue HalA. An HMMTOP (www.enzim.hu/hmmtop) prediction is that TrkB has eight trans-membrane (TM) domains and that Ser372 is in a highly conserved cytosolic loop between TM2 and TM3. The effect of *trkB1* on Li^+^ efflux appears novel; to our knowledge, no such role has been reported for a *TRK1* or *TRK2* allele.

Selection for suppression of the calcium auxotrophy could easily yield numerous additional *nhaA* mutant alleles, enabling an extensive mutational dissection to accompany a biochemical and physiological analysis. An earlier report (Kamauchi et al., 2002) suggested that *A. nidulans* NhaA contains 12 TM domains. Various TM prediction sites predict between 10 and 13 TMs for NhaA and between 9 and 13 TMs for *S. cerevisiae* Nha1p. Fig. 8 shows our sequenced mutations placed on an HMMTOP prediction in which NhaA contains 13 TM domains with the long hydrophilic C-terminus located inside. Amongst our eight sequenced mutations are single residue substitutions in predicted TM4, TM5, TM10 and TM12 and in the predicted 12 residue internal loop between TM11 and TM12 as well as a truncation almost immediately after TM13, removing 58.5% of the protein, comprising the poorly conserved, predicted cytosolic C-terminal region (Fig. 8). It is worth noting that the C-terminal moiety of *S. cerevisiae* Nha1p is essential to function (Kamauchi et al., 2002; Mitsui et al., 2004).

### 4.3 The calcium auxotrophy

**Fig. 9:**
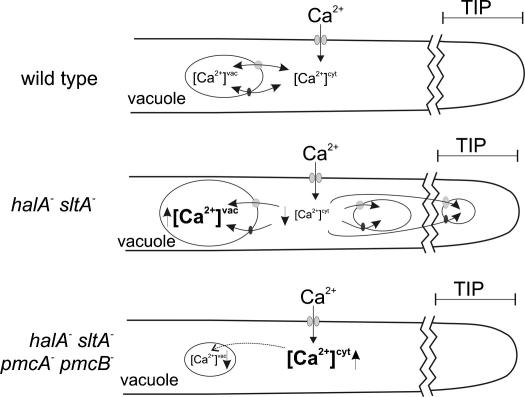
Model showing vacuolar system hypertrophy and excessive vacuolar Ca^2+^ sequestration in *halA*^−^*sltA*^−^ double mutants and vacuolar fragmentation with defective vacuolar Ca^2+^ sequestration in *halA*^−^*sltA*^−^*pmcA*^−^*pmcB*^−^ quadruple mutants.

The *halA*^−^ *sltA*^−^ elevated calcium requirement or ‘calcium auxotrophy’ of *A. nidulans* is a valuable new tool for investigating calcium signalling and homeostasis, enabling the selection and physiological characterisation of mutations in calcium-related genes. The elevated calcium requirement and the new information on the phenotypes of *halA* and *sltA* mutations are also of interest in the context of intracellular cation distribution and homeostasis. Of possible relevance to this work are the findings that loss of the major isoform of phosphoglucomutase in *S.* *cerevisiae* combined with transfer of the mutant to galactose medium results in excessive calcium uptake as a result of *PMC1* over-expression and that deletion of *PMC1* improves growth of the phosphoglucomutase-less mutant on galactose (Aiello et al., 2004). In *A.* *nidulans*, the vacuolar system hypertrophy as a consequence of *sltA*^−^ mutations, the strikingly elevated transcript levels for *pmcA* and *pmcB* in *halA*^−^ *sltA*^−^ double mutants, the ability of combined deletion of *pmcA* and *pmcB* to alleviate the calcium auxotrophy and the calcium sensitivity of *pmcA pmcB* double deletants argue that excessive vacuolar sequestration of calcium and the consequent cytosolic deprivation are responsible for the elevation in calcium requirement. Our hypothesis for the basis of the calcium auxotrophy is modelled in Fig. 9. The idea that a mutation(s) leading to vacuolar hypertrophy and over-expression of vacuolar calcium pumps constitutes a means for depriving the cytosol of calcium by excessive vacuolar storage, enabling manipulation of intracellular calcium, is one of possibly widespread utility.
