# Supplementary material for: Analysis of a novel calcium auxotrophy in Aspergillus nidulans
Source: Fungal Genet Biol. 2010 Jul;47(7):647–55. doi: 10.1016/j.fgb.2010.04.002 (PMC2884188; doi:10.1016/j.fgb.2010.04.002)
Supplement: Supplementary material [file mmc1.doc]

**Supplementary Table S1. Oligonucleotides used in this work**

| Name | location | Sequence 5’ to 3’ |
| --- | --- | --- |
| 5’ AfPyroA 1189 | Selectable marker fw | TCTTTGAGGCCCGTCATCGGACATCAGATGCTGGATTA |
| 3’ AfPyroA 1189 | Selectable marker rev | TTGATTGAGACTGAGGAGCGTATCACAATCAGCTTTTCAGA |
| AN1189 5’5’ | 5’ UTR rev | TGGCGCAGGTTTCGTATT |
| 5’3’ AfPyroA 1189 | 5’ UTR fw | TAATCCAGCATCTGATGTCCGATGACGGGCCTCAAAGA |
| AN1189 3’3’ | 3’ UTR rev | TTGCGGCAGAGATATACCTGT |
| 3’5’ AfPyroA 1189 | 3’ UTR fw | TCTGAAAAGCTGATTGTGATACGCTCCTCAGTCTCAATCAA |
| 5’AfPabaA 4920 | Selectable marker fw | TTTACTACGCGGCAGTTCGTCAAAATGTTCCGTTGATTGCC |
| 3’AfPabaA 4920 | Selectable marker rev | TGAGAACGCCAAAGTCAAGTTCAAAATTGTCTTCGCAGGG |
| AN4920 5’5’ | 5’ UTR rev | AACCACGTGCGAGGCATATT |
| 5’3’ AfPabaA 4920 | 5’ UTR fw | GGCAATCAACGGAACATTTTGACGAACTGCCGCGTAGTAAA |
| AN4920 3’3’ | 3’ UTR rev | CCGCCATACCCTTGTACCTAA |
| 3’5’ AfPabaA 4920 | 3’ UTR fw | CCCTGCGAAGACAATTTTGAACTTGACTTTGGCGTTCTCA |
| VcxA-GFP2 | Selectable marker rev | GAATATATCGAGCAGCCAGCAGCATAGTGTGTCTGAGAGGAGGCACTGATGCG |
| VcxA-SMP1 | Selectable marker fw | CTACGCGATCGGACAGCATCAACAAGTATCACCGGTCGCCTCAAACAATGCTCT |
| VcxA PP2 | 5’ UTR rev | GATACTTGTTGATGCTGTCCGATCGCGTAG |
| VcxA-rev | 3’ UTR rev | GAGGAGCGGAGGATGTCGAGGATTATGTTC |
| VcxA-GSP3 | 3’ UTR fw | ACACTATGCTGCTGGCTGCTCGATATATTC |
| VcxA-fw | 5’ UTR fw | TCAGCCAAGGTTATGCACCTTCACTATCC |
| TrkB-SMP1 | Selectable marker rev | CAAGTCCACACCACTTCTCGTCCTGGTCCGACCGGTCGCCTCAAACAATGCTCT |
| TrkB-GFP2 | Selectable marker fw | GTTGGAGTTGTGTCGGCCTCGTGAGCGTAAGTCTGAGAGGAGGCACTGATGCG |
| TrkB-promfw | 5’ UTR fw | ATTCCACCACGGCGAGCTGCTGAATAAGG |
| TrkB-promrev | 5’ UTR rev | CGGACCAGGACGAGAAGTGGTGTGGACTTG |
| TrkB-termfw | 3’ UTR fw | TTACGCTCACGAGGCCGACACAACTCCAAC |
| TrkB-termrev | 3’ UTR rev | ACGCCTCCATTAGTCGCGAGAGGAGACAGA |
| F1-promPep12 | 5’ UTR fw | GCGGCCGCAGTAGGCGCAGACCACTTGT |
| R1-promPep12 | 5’ UTR rev | ACTCCAGTGAAAAGTTCTTCTCCTTTACTCATATTGTAGCGAGAGACGCAGC |
| F2-GFP | GFP fw | ATGAGTAAAGGAGAAGAACTTTTCACTG |
| R2-GA5-GFP | GFP and linker rev | GGCACCGGCTCCAGCGCCTGCACCAGCTCCTTTGTATAGTTCATCCATGC |
| F3-GA5-Pep12 | linker and p*epA* (+4 to +24 bp) fw | GGAGCTGGTGCAGGCGCTGGAGCCGGTGCCTCTTTCAATCACTTGAGCTC |
| R3-Pep12-AfPyrG | *pepA* (+888 to +868 bp) rev | GCGAAGAGGGTGAAGAGCATTGTTTGAGGCCCTTGGAAGCAGGAGTTTCG |
| F4-Pep12-PyrGf | Selectable marker fw | CAACCGATACCGAAACTCCTGCTTCCAAGGGCCTCAAACAATGCTCTTCA |
| R4-Pep12-PyrGf | Selectable marker rev | GGAACTCTGTTCAAATTTCTCGCGGTATGACTGATGCGTGATGCCAAGCT |
| F5-downPep12 | 3’ UTR fw | AAGAGATAACAGCTTGGCATCACGCATCAGTCATACCGCGAGAAATTTGA |
| R5-downPep12 | 3’ UTR rev | GCGGCCGCCATTACCAAGCACCACAACG |
| 5’AfPabaA TrkA | Selectable marker fw | TAAAGCTTCTGCCGGGAAATCAAAATGTTCCGTTGATTGCC |
| 3’AfPabaA TrkA | Selectable marker rev | CCAGAAGTGCATTCATTCCATCAAAATTGTCTTCGCAGGG |
| TrkA 5’5’ | 5’ UTR rev | CCCGTTCCAGAGTAAGATGAT |
| 5’3’ AfPabaA TrkA | 5’ UTR fw | GGCAATCAACGGAACATTTTGATTTCCCGGCAGAAGCTTTA |
| TrkA 3’3’ | 3’ UTR rev | AAGCAGCACATACCGAAGAGA |
| 3’5’ AfPabaA TrkA | 3’ UTR fw | CCCTGCGAAGACAATTTTGATGGAATGAATGCACTTCTGG |
| AN8029.3-TrkB For | *trkB* (+2193 to + 2210 bp)fw | TGGGCTGCCGTACTCTCT |
| AN8029.3-TrkB Rev | *trkB* (+2246 to + 2269 bp)rev | CTGCGTCCAAGATTTCTTTTTGCT |
| AN8029.3-TrkB FAM | *trkB* (+2213 to + 2226bp) rev | ACCGCGCCATTTTG |
| AN1189.3-PmcA For | *pmcA* (+243 to + 265 bp) fw | AGCTCATTCAGTACGCTCGTATG |
| AN1189.3-PmcA Rev | *pmcA* (+283 to + 302 bp) rev | GTTGGCCTGCTATCGTGTTC |
| AN1189.3-PmcA FAM | *pmcA* (+267 to + 282 bp) rev | CCTGCCCTCTGACGAA |
| AN4920.3-PmcB For | *pmcB* (+3438 to + 3459 bp) fw | CGATGCGAACGCTTTGACATAT |
| AN4920.3-PmcB Rev | *pmcB* (+3485 to +3501 bp) rev | CACGGAGCGCTGTTCCT |
| AN4920.3-PmcB FAM | *pmcB* (+3460 to +3475 bp) rev | CACGCTGTTCCTTTAG |
| AB008403-18S For | 18S rRNA Normalizing control fw | GCCTTTGCTCGGATACATTAGC |
| AB008403-18S Rev | 18S rRNA  Normalizing control rev | GCGGTCCTAGAAACCAACAAAATAG |
| AB008403-18S FAM | 18S rRNA  Normalizing control rev | CCGCACGTCCTATTC |

The last 12 oligonucleotides listed are those used for RTQ-PCR.
