# Supplementary material for: Analysis of a novel calcium auxotrophy in Aspergillus nidulans
Source: Fungal Genet Biol. 2010 Jul;47(7):647–55. doi: 10.1016/j.fgb.2010.04.002 (PMC2884188; doi:10.1016/j.fgb.2010.04.002)
Supplement: Supplementary material [file mmc2.doc]

**Supplementary Table S2. Strains used in displayed experiments.**

| Strain designation | Full genotype | Figure(s) |
| --- | --- | --- |
| 2836e | *pabaA1 yA2* | 1A, 3A-B, 4, 7A-C |
| 2836c | *pabaA1 yA2 halAΔ::pyr-4 sltAΔ::riboB^f^* | 1A, 3A-B, 4,  7A |
| 2836b | *pabaA1 yA2 halAΔ::pyr-4* | 1A, 3A-B, 4 |
| 2836a | *pabaA1 yA2 sltAΔ::riboB^f^* | 1A, 3A-B, 4 |
| MAD1336 | *biA1 phenA2 cbxA17* | 1B |
| MAD1337 | *halA24 inoB2 fwA1* | 1B |
| MAD1131 | *yA2 inoB2 sltA1* | 1B |
| MAD1057 | *biA1 pabaA1 yA2 halA24 sltA1* | 1B |
| AMC289 | *pabaA1 yA2 gfp::pepA::pyrG^f^ sltAΔ::riboB^f^* | 2A |
| AMC346 | *biA1 gfp::pepA::pyrG^f^ pyroA4 pantoB100* | 2A |
| MAD1046 | *pabaA1 wA2 pyroA4* | 2B |
| AMC207 | *pabaA1 pyroA4 sltA54* | 2B |
| 2542c | *wA3 halA24 sltA1 glrA1 pantoB100* | 4 |
| A131 | *biA1 halA24 pmcBΔ::pabaA^f^ sltA1 pantoB100 pmcAΔ::pyroA^f^* | 4, 6A |
| A123 | *biA1 wA3 pmcBΔ::pabaA^f^ pmcAΔ::pyroA^f^* | 4, 6B |
| HHF27a | Prototrophic wild type | 5 |
| HHF27b | *sltAΔ::riboB^f^* | 5 |
| HHF27c | *halAΔ::pyr-4* | 5 |
| HHF27d | *halAΔ::pyr-4 sltAΔ::riboB^f^* | 5 |
| A149 | *wA3 pantoB100* | 6A |
| A95 | *wA3 halA24 sltA1 pantoB100* | 6A |
| A134 | *wA3 halA24 sltA1 pantoB100 pmcAΔ::pyroA^f^* | 6A |
| A127 | *biA1 wA3 halA24 pmcBΔ::pabaA^f^ sltA1 pantoB100* | 6A |
| A148 | *pabaA1 wA3* | 6B |
| HHF45g | *biA1 pmcBΔ::pabaA^f^* | 6B |
| A121 | *biA1 wA3 pmcAΔ::pyroA^f^* | 6B |
| A98 | *trkB1 halAΔ::pyr-4 inoB2 sltAΔ::riboB^f^* | 7A |
| A99 | *halAΔ::pyr-4 inoB2 nhaA1 sltAΔ::riboB^f^* | 7A |
| 2907c | *biA1 pyroA4 nhaA1* | 7B-C |
| 2907i | *pabaA1 adH23 trkB1* | 7B-C |
